# New Solutions of Fractional Jeffrey Fluid with Ternary Nanoparticles Approach

**DOI:** 10.3390/mi13111963

**Published:** 2022-11-12

**Authors:** Muhammad Imran Asjad, Ayesha Riaz, Abeer S. Alnahdi, Sayed M. Eldin

**Affiliations:** 1 Department of Mathematics, University of Management and Technology, Lahore 54770, Pakistan; 2 Department of Mathematics and Statistics, Faculty of Science, Imam Mohammad Ibn Saud Islamic University, Riyadh 11623, Saudi Arabia email; 3 Center of Research, Faculty of Engineering and Technology, Future University in Egypt, New Cairo 11835, Egypt

**Keywords:** ternary nanoparticles, hybrid nanoparticles, mono nanoparticles, Jeffrey fluid, Prabhakar fractional operator

## Abstract

The existing work deals with the Jeffrey fluid having an unsteady flow, which is moving along a vertical plate. A fractional model with ternary, hybrid, and nanoparticles is obtained. Using suitable dimensionless parameters, the equations for energy, momentum, and Fourier’s law were converted into non-dimensional equations. In order to obtain a fractional model, a fractional operator known as the Prabhakar operator is used. To find a generalized solution for temperature as well as a velocity field, the Laplace transform is used. With the help of graphs, the impact of various parameters on velocity as well as temperature distribution is obtained. As a result, it is noted that ternary nanoparticles approach can be used to increase the temperature than the results obtained in the recent existing literature. The obtained solutions are also useful in the sense of choosing base fluids (water, kerosene and engine oil) for nanoparticles to achieved the desired results. Further, by finding the specific value of fractional parameters, the thermal and boundary layers can be controlled for different times. Such a fractional approach is very helpful in handling the experimental data by using theoretical information. Moreover, the rate of heat transfer for ternary nanoparticles is greater in comparison to hybrid and mono nanoparticles. For large values of fractional parameters, the rate of heat transfer decreases while skin friction increases. Finally, the present results are the improvement of the results that have already been published recently in the existing literature. Fractional calculus enables us to control the boundary layers as well as rate of heat transfer and skin friction for finding suitable values of fractional parameters. This approach can be very helpful in electronic devices and industrial heat management system.

## 1. Introduction

The fluid is a type of matter that is constantly distorted when an insignificant quantity of force is applied outwardly [1]. Non-Newtonian fluids in comparison to Newtonian fluids have become more suitable for scientific as well as technical disciplines. Non-Newtonian fluids have created a lot of interest in the field of engineering as well as in science because of their massive range of industrial applications, food processing, lubricant performance and many more. Fluids not obeying Newton’s law are termed as non-Newtonian fluids. In order to define the viscous behavior of these kinds of fluids, numerous methods were suggested. A subclass of these fluids, specifically, Jeffrey fluid, attained much interest in recent years due to the fact that the constitutive equation for Jeffrey can be condensed to that of the Newtonian model. The effects of retardation and also relaxation are demonstrated with the help of the Jeffrey fluid [2,3,4]. Jeffrey is not only a basic theoretical idea, but is also used to explain various practical problems [5]. In peristalsis, Jeffrey fluid has been also considered as the main source of fluid transport, particularly in a biological system. There are some similar efforts for the Jeffrey fluid in [6,7,8].

Fractional calculus extends the integer order to fractional order calculus by means of providing the opportunity of taking fractional derivatives as well as integrals [9]. The fractional calculus approach is beneficial on the subject of generalization of complex dynamics of fluid movement [10]. Fractional calculus is growing vastly nowadays and is gaining attention due to its multipurpose and sole properties. Fractional calculus is a significant tool for telling numerous systems counting memory. Fractional calculus has been utilized for numerous purposes in several areas, for example, electrochemistry, electromagnetism, diffusion, conduction of heat process, transportation, and elasticity in recent times [11]. Numerous models of physical progressions have been efficiently altered by mean of fractional calculus [12]. In the last few years, the idea of utilizing fractional calculus in making constitutive equations for constituents with a memory effect has been projected several times [13]. The necessity for Prabhakar operators to have precise fractional coefficients might be valuable for defining statistical techniques, which formed strong agreement among theoretical as well as experimental results [14]. The Prabhakar integral is attained through the outspreading kernel of the Riemann–Liouville integral with a three-parameter Mittag–Leffler function [15]. For the past several years, numerous studies have been focused on using nanofluids [16]. The applications of nanofluids have gained enormous interest because they have relatively improved heat transfer features [17]. Nanofluids usage in numerous fields is rising. Some examples of its applications include solar thermal systems [18], cooling of electronic devices, and heat exchangers [19,20]. The thought of mixing nanoparticles by common base fluids to improve thermal performance and thermophysical characteristics of conventional fluids were recommended for the first time in 1995 [21]. Nanofluids contain the dispersion of nanoscale particles in conventional heat transfer fluids. Overall, these particles comprise metals, carbon-based essentials, and metal oxides. Many researchers illustrated that the thermal properties of a fluid increase after the addition of nanoparticles to the fluid [22]. Nanoparticles can swap conventional materials and help as a material of support and heterogeneous catalytic systems for unalike catalytic systems [23]. Nanomaterials are widely used in the field of biology or medicine such as in fluorescent biological labels, tissue engineering, detection of proteins, MRI contrast enhancement, etc. [24]. Nanoparticle formulations are applied for numerous purposes and they can be used as therapeutics and cancer diagnostics. Most nanoparticles are considered perfect aspirants to image the MPS, which includes the spleen, lymphatics, and liver [25].

Nanofluids contain metal oxide elements suspended in the alkaline fluid, which enhance the characteristics like conductivity and convective heat transfer in any base fluid. The thermal conductivity of solid atoms is usually better than that of base fluids; even at inferior concentrations, the thermal performance is meaningfully enhanced. Additionally, the nanofluids help in declining their heat resistance, and industries can obtain an advantage from the altered nanofluids containing heat transfer properties [26]. The blend of two or more than two nanoparticles in a base fluid is called a hybrid nanofluid. Viscosity is the flow measurement of nanofluids. It is vital for comparing the thermo-fluid behavior of heat transfer fluids, additionally its outcomes because of the heat transfer with the effect of atmospheric air when traveling the streamline flow [27]. Hybrid nanofluids are more beneficial as compared to traditional fluids and nanofluids due to their outstanding performance. Major factors which disturb the performance of hybrid nanofluids are ultrasound intensity, stability, pH control, thermal conductivity, frictional factor, and electrical conductivity. The dominance of hybrid nanofluids over base fluids and nanofluids makes them the primary choice to be used extensively in the engineering as well as industrial sectors [28]. No precise investigation has been completed on thermophysical and heat transfer development by means of metal oxide-based original ternary hybrid complex nanofluids [29]. The formation of three-particle hybrid nanofluids named as ternary hybrid nanofluids has newly resentful researcher’s interest. Similar to how hybrid nanofluids are supposed to have better thermophysical properties because of synergistic effects, ternary nanofluids are improved heat transfer fluids in experimental research [30]. The changing aspects of ternary hybrid nanofluids fit the precise explanation of Newtonian fluid. Due to increased temperature, the density of ternary hybrid nanofluids decreases linearly [31]. For industrial use, ternary hybrid nanofluids are vital to select. Scientists appreciate how the thermophysical properties and rheological behavior are affected by various parameters like temperature, mixture ratio, and volume fraction of such nanofluids [32]. Saleem et al. inspected the changing aspects of ternary-hybrid nanofluids with an accent on the variation of heat, friction, and mass transfer rates crossways the domain. The growth of species that shape water-based ternary-hybrid nanofluids is declining because of the rising unpredictable motion and thermomigration of diverse nanoparticles [33]. In thermal systems, the use of ternary hybrid nanoparticles have is the least acknowledged and all the earlier numerical and experimental research mainly focused on binary or mono nanofluids [34]. In this paper, Yb-doped and pure and ZnO [Zn1-xYbxO (x = 0, 0.01, 0.03, 0.05)] gels were set via the sol-gel procedure and then they were screen-printed on a glass substrate; as a result, UV visible spectroscopy (300–800 nm) proves that ZnO film demonstrates practical transparency in the visible range, having a supreme transmission of 60 percent, and the band gap diminishes from 3.29 to 3.22 eV by using Yb- doping [35]. In the previous two years, academicians have focused on the nanofluid squeezing flow in between the parallel plates. The applications of nanofluids have seen the attention shifted to the hybrid nanofluid, but still, there is so much to be investigated. This examination is performed to explore the symmetry of MHD squeezing hybrid nanofluid ($MoS_{2}$–$SiO_{2}$/$H_{2}$O–$C_{2}H_{6}O_{2}$) flow and nanofluid ($MoS_{2}$/$H_{2}O$) flow between the parallel plates. The consequence shows that at the bottom of the plate, the Nusselt number is greater for the ($MoS_{2}$–$SiO_{2}$/$H_{2}O$–$C_{2}H_{6}O_{2}$) hybrid nanofluid as compared to the ($MoS_{2}$/$H_{2}O$) nanofluid flow [36].

The Falkner–Skan problem for stretching is comprehensive for nanoparticle aggregation effects. The model is made in the occurrence of injection/suction effects, thermal radiation, and magnetic field. It is seen that the effect of nanoparticle aggregation is durable even when the concentration of nanoparticles is less. The $TiO_{2}$/EG nanofluid heat transmission rate is found to be higher with nanoparticle aggregation effects in contrast to when it is absent [37]. In this, magnetic ferrite nanoparticles were manufactured and glazed with silica $\left(ferrite@SiO_{2}NPs\right)$ by means of a sol-gel method. Afterward, silica propylmethylimidazolium chloride ionic liquid [Sipmim]Cl was equipped and related with the above-equipped ferrite$@SiO_{2}NPs$ to manufacture ferrite silica propylmethylimidazolium chloride [Fesipmim]Cl catalyst [38]. The foremost focus of this examination is to reveal the mathematical modeling of chemically reactive Maxwell nanofluid for axisymmetric flow case by seeing Cattaneo–Christo heat flux model and reviewed nanofluid model. The result shows that associated boundary layer thickness and temperature produces due to the enrichment of the heat source and temperature ratio parameter. Moreover, advanced approximation of radiation and magnetic parameter shows devaluation of wall heat transfer. Additionally, the concentration of nanoparticles is disparaged for raising the values of reaction rate parameters [39]. Azam et al. presented a mathematical demonstration and numerical solution for unsteady Bioconvection flow having chemically sensitive Sutterby nanofluid under the impact of nonlinear radiation and gyrotactic microorganism. The results obtained are very interesting, as it is noted that the microorganism field is elevated for advanced estimation of the microorganism alteration parameter and Peclet number. Furthermore, the greater bioconvection Rayleigh number disparages the fluid velocity [40]. Azam et al. also devise novel mathematical models for an unsteady bioconvection flow of chemically responsive Casson nanoliquid with gyrotactic microorganisms and non-linear for axisymmetric flow case. Additionally, viscous dissipation ramifications as well as activation energy are observed. It is observed that the density number for microorganisms is enounced for advanced approximation of the microorganism difference parameter and Peclet number [41]. Azam et al. proposed a model of transient bioconvection of Maxwell nanofluid under the inferences of variable thermal conductivity and viscosity for the case of axisymmetric flow and gyrotactic microorganisms. It is found that the microorganism density number is wired because of the rising bioconvection Lewis number and Peclet number, but the opposite behavior is dejected for a strengthened Maxwell parameter [42]. Azam et al. investigate numerical exploration concerning the bioconvection ramifications and activation energy in the growth of thermal extrusion of a chemically delicate Casson nanoliquid over a holey cylinder under the consequences of viscous dissipation and gyrotactic microorganism. The consequences of this study disclose that the density number of microorganisms is deplored for advanced approximation of the microorganism difference parameter and Peclet number. Additionally, wall heat flux is belittled due to the adulation of the Rayleigh number [43]. In light of the above recent literature, researchers have examined applications of different types of nanoparticles and found the solutions for different geometries and fluids of Newtonian and non-Newtonian nature. In the above mentioned literature, researchers have ignored the fractional approach of such models. The main advantage of the fractional approach can also help us to improve the fluid properties for various values of fractional parameters and times. Usually, the mentioned models in the introduction are represented in terms of traditional partial differential equations (PDEs), and these PDEs are unable to decode the complex behavior of physical flow parameters and memory effects. The classical calculus only measures the instant rate of change of the output, when the input level changes. Therefore, it is not able to include the previous state of the system called memory effect. On the other hand, in fractional calculus, the rate of change is affected by all the points of the considered interval, so it is able to incorporate the previous history/memory effects of any system. Fractional calculus enables us to control the boundary layers as well as the rate of heat transfer and skin friction for finding suitable value of fractional parameter. This approach can be very helpful in electronic devices and industrial heat management systems. The thermophysical properties of nanofluids are given in Table 1.

**Research questions**
How to do fractional modeling in heat transfer?
How is the rate of heat transfer of ternary nanoparticles greater than hybrid and mono nanoparticles, respectively?
What is the advantage of choosing different base fluids for three types of nanoparticles?

## 2. Mathematical Formulation

**Figure 1:**
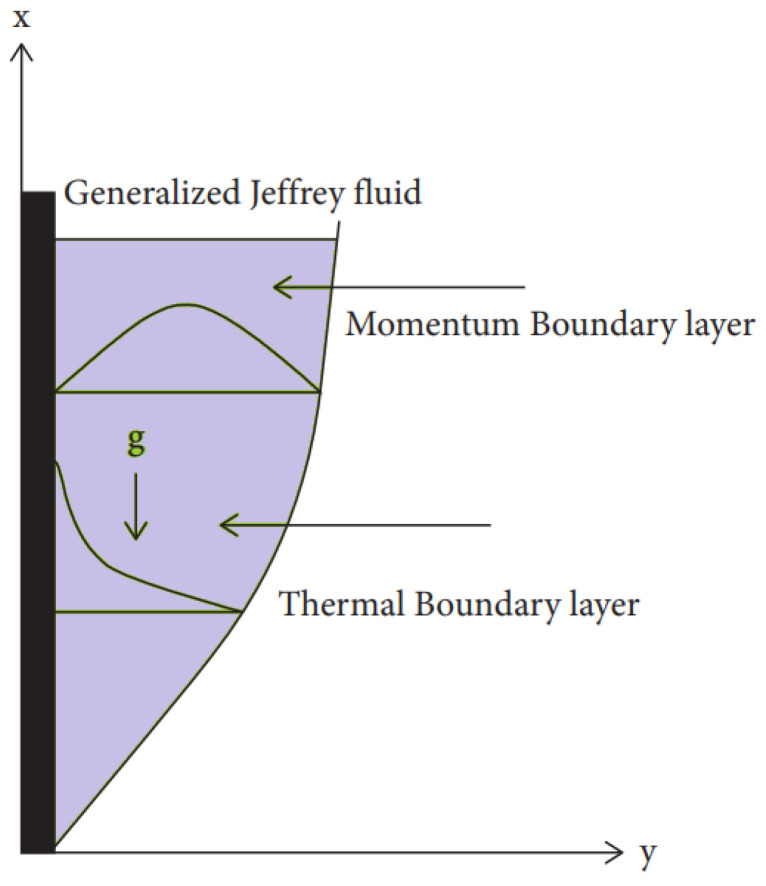
Model geometry.

Let us consider the unsteady flow of Jeffrey fluid containing ternary nanoparticles over an infinite vertical plate situated at y=0 along the *x*-direction as shown in the Figure 1. At the beginning, both the plate and the nanofluid are stationary with constant wall temperature $T_{\infty }$. When t>0, the plate remains at rest while the temperature is raised to $T_{w}$. Under the Boussinesq approximation, temperature and velocity are the functions of *y* and *t* only, and the governing equations for Jeffrey fluid containing ternary nanoparticles are given as [2].

(1) T=S−Ip,

(2) $$S=\frac{\mu }{1+\lambda }\left(A+\lambda_{1}\frac{dA}{dt}\right),$$

(3) $$\frac{d}{dt}=\frac{\partial }{\partial t}+\nabla .V,$$

where *S* is the extra stress tensor, $\lambda_{1}$ and λ are material parameters and -pI is indeterminate part of the Jeffrey fluid.

Rivlin–Ericksen tensor is

(4) $$A=L^{T}+L,$$

L=∇V.

The velocity, stress, and temperature fields for one-dimensional and unidirectional flow is

$$V=\bar{u}\left(\bar{t},\bar{y}\right)\hat{i},$$

(5) $$S=S(\bar{t},\bar{y}),$$

$$T=\bar{T}\left(\bar{t},\bar{y}\right),$$

$\hat{i}$ indicates unit vector in x-direction and the stress field satisfying the constraints *S*($\bar{y},0$). From this, we obtain $S_{xx}$ = $S_{xz}$ = $S_{zx}$ = 0, $S_{yy}$ = $S_{yz}$ = 0, $S_{zz}$ = $S_{zy}$ = 0 and

$$S_{xy}=\frac{\mu }{1+\lambda }\frac{\partial \bar{u}}{\partial \bar{y}}\left(1+\lambda_{1}\frac{\partial }{\partial \bar{t}}\right),$$

where $S_{xy}$ indicates non-trivial tangential stress. By means of Equations (1)–(5) and Boussinesq approximation equation of momentum, energy, and also Fourier’s law are found in [13].

## 3. Ternary Nanoparticles

Momentum equation for **ternary nanoparticles** [2].

(6) $$\rho_{mnf}\frac{\partial \bar{u}}{\partial \bar{t}}=\frac{\mu_{mnf}}{1+\lambda }\left(1+\lambda_{1}\frac{\partial }{\partial \bar{t}}\right)\frac{\partial^{2}\bar{u}}{\partial {\bar{y}}^{2}}+g{\left(\rho \beta_{T}\right)}_{mnf}\left[\bar{T}-T_{\infty }\right].$$

Energy equation for ternary nanoparticles [2].

(7) $${\left(\rho C_{p}\right)}_{mnf}\frac{\partial \bar{T}}{\partial \bar{t}}=\frac{\partial \bar{q}}{\partial \bar{y}}.$$

Generalized Fourier law [2].

(8) $$\bar{q}=-k_{mnf}^{C}D_{\alpha ,\beta ,a}^{\gamma }\bar{T}\bar{y}.$$

The boundary constraints for temperature and velocity are as follows:

(9) $$for\;\;\bar{u}\left(y,0\right)=0,\;\;\;\;\;\bar{T}\left(y,0\right)=T_{\infty },\;\;\;t\leq 0,$$

(10) $$for\;\;\bar{u}\left(0,t\right)=0,\;\;\;\;\;\bar{T}\left(0,t\right)=T_{w},\;\;\;t>0,$$

(11) $$\bar{u}\left(y,t\right)\to 0,\;\;\;\;\;\bar{T}\left(y,t\right)\to T_{\infty },\;\;\;as\;\;\;y\to \infty .$$

where $\rho_{mnf}$, $\mu_{mnf}$, ${\left(\rho \beta_{T}\right)}_{mnf}$ and ${\left(\rho C_{p}\right)}_{mnf}$ and $k_{mnf}$ are density, viscosity, volumetric expansion, specific heat transfer and thermal conductivity for ternary nanoparticles. The properties of nanoparticles are defined in Table 2 [12].

In order to obtain the problem free from the flow region, some dimensionless variables are introduced from Equation (12):

(12) $$y=\frac{u_{0}}{\nu }\bar{y},\;t=\frac{u_{0}^{2}}{\nu }\bar{t},\;u=\frac{\bar{u}}{u_{0}},\;\theta =\frac{\bar{T}-T_{\infty }}{T_{w}-T_{\infty }},\;q=\frac{\bar{q}}{q_{0}},\;q_{0}=\frac{k\left(T_{w}-T_{\infty }\right)\mu_{0}}{\nu }$$

into (6)–(11), we have dimensionless governing equations:

(13) $$b_{1}\frac{\partial u}{\partial t}=\frac{b_{2}}{1+\lambda }\left(1+\lambda_{2}\frac{\partial }{\partial t}\right)\frac{\partial^{2}u}{\partial y^{2}}+\mathrm{Gr}\theta b_{3},$$

(14) $$\frac{\partial \theta }{\partial t}b_{0}=\frac{1}{\mathrm{Pr}}\frac{\partial q}{\partial y},$$

(15) $$q=-{\;}^{C}D_{\alpha ,\beta ,a}^{\gamma }\theta ,$$

where $\mathrm{Pr}=\frac{\mu C_{p}}{k}$ is a dimensionless Prandtl number, $\mathrm{Gr}=\frac{(T_{w}-T_{\infty })g\nu \beta }{u_{o}^{3}}$ is a dimensionless thermal Grashof number and $\lambda_{2}=\left(\lambda_{1}\frac{u_{0}^{2}}{\nu }\right)$ are Jeffrey parameters, respectively.

Dimensionless constraints are as follows:

(16) t=0,u=0,θ=0,

(17) y=0,u=0,θ=1,

(18) y→∞,u→0,θ→0.

where

$b_{0}=\left[\left(1-\left(\phi_{1}+\phi_{2}+\phi_{3}\right)\right)+\frac{\phi_{1}{\left(\rho C_{p}\right)}_{s_{1}}+\phi_{2}{\left(\rho C_{p}\right)}_{s_{2}}+\phi_{3}{\left(\rho C_{p}\right)}_{s_{3}}}{{\left(\rho C_{p}\right)}_{f}}\right]$,

$b_{1}=\left[\left(1-\left(\phi_{1}+\phi_{2}+\phi_{3}\right)\right)+\frac{\phi_{1}\rho_{s_{1}}+\phi_{2}\rho_{s_{2}}+\phi_{3}\rho_{s_{3}}}{\rho_{f}}\right]$,

$b_{2}=\frac{1}{{\left(1-\left(\phi_{1}+\phi_{2}+\phi_{3}\right)\right)}^{2.5}}$,

$b_{3}=\left[\left(1-\left(\phi_{1}+\phi_{2}+\phi_{3}\right)\right)+\frac{\phi_{1}{\left(\rho \beta_{T}\right)}_{s_{1}}+\phi_{2}{\left(\rho \beta_{T}\right)}_{s_{2}}+\phi_{3}{\left(\rho \beta_{T}\right)}_{s_{3}}}{\left(\rho \beta_{T}\right)}\right].$

### 3.1. Solution of Temperature with Ternary Nanoparticles

Applying Laplace transform on Equations (14) and (15) with boundary conditions of (16) and using Prabhakar fractional derivative. The temperature field is

(19) $$s\bar{\theta }\left(y,s\right)b_{0}+\frac{1}{\mathrm{Pr}}\frac{\partial \bar{q}}{\partial y}\left(y,s\right)=0,$$

(20) $$\bar{q}\left(y,s\right)=-{\left(1-as^{-\alpha }\right)}^{\gamma }s^{\beta }\frac{\partial \bar{\theta }}{\partial y},$$

(21) $$\bar{\theta }\left(0,s\right)=\frac{1}{s},\;\;\;\;\;\bar{\theta }\left(y,s\right)\to 0,\;\;\;\;\;as\;\;\;\;y\to \infty$$

Introducing Equation (20) into (19):

(22) $$\frac{\partial^{2}\bar{\theta }}{\partial y^{2}}\left(y,s\right)-\frac{\mathrm{Pr}sb_{0}}{s^{\beta }{\left(1-as^{-\alpha }\right)}^{\gamma }}\bar{\theta }\left(y,s\right)=0,$$

The general solution of Equation (22), which satisfies the boundary constraints given in Equation (21), is

(23) $$\bar{\theta }\left(y,s\right)=s^{-1}e^{-y\sqrt{\frac{\mathrm{Pr}sb_{0}}{s^{\beta }{\left(1-as^{-\alpha }\right)}^{\gamma }}}}.$$

To obtain inverse Laplace transform, Equation (23) can be written in the following form by using formula ${\left(1-x\right)}^{-p}=\sum_{q=0}^{\infty }x^{q}\left(\frac{\Gamma (p+q)}{q!}\left(\Gamma p\right)\right)$ [2].

(24) $$\bar{\theta }\left(y,s\right)=\frac{1}{s}+\sum_{m=1}^{\infty }\sum_{n=0}^{\infty }\frac{{\left(-y\right)}^{m}{\left(\mathrm{Pr}b_{0}\right)}^{\frac{m}{2}}}{m!\;s^{\frac{\beta m-m}{2}+\alpha a+1}}\frac{{\left(-a\right)}^{n}}{n!}\frac{\Gamma (\frac{-\gamma m}{2}+1)}{\Gamma (\frac{-\gamma m}{2}+1-n)}.$$

The inverse Laplace of Equation (24) by using formula $L^{-1}\left\{\frac{1}{s^{n}}\right\}=\frac{t^{n-1}}{\Gamma (n)}\;\;\;n\geq 0.$

(25) $$\theta \left(y,t\right)=1+\sum_{m=1}^{\infty }\sum_{n=0}^{\infty }\frac{{\left(-y\right)}^{m}{\left(\mathrm{Pr}b_{0}\right)}^{\frac{m}{2}}}{m!}\frac{t^{\frac{\beta m-m}{2}+\alpha a}}{\Gamma (\frac{\beta m-m}{2}+\alpha a+1)}\frac{{\left(-a\right)}^{n}}{n!}\frac{\Gamma (\frac{-\gamma m}{2}+1)}{\Gamma (\frac{-\gamma m}{2}+1-n)}.$$

### 3.2. Solution of Velocity with Ternary Nanoparticles

Using Laplace transform on Equations (6), (10) and (11) and utilizing Prabhakar fractional derivative.

(26) $$b_{1}s\bar{u}\left(y,s\right)=\frac{b_{2}}{1+\lambda }\left(1+\lambda_{2}s\right)\frac{\partial^{2}\bar{u}}{\partial y^{2}}+\mathrm{Gr}\bar{\theta }b_{3},$$

(27) $$\bar{u}\left(y,s\right)=0,\;\;\;\;\;\;\;\bar{u}\left(0,s\right)=0.$$

The solution of Equation (26) satisfying boundary conditions (27) is

(28) $$-\mathrm{Gr}\bar{\theta }\;\;\frac{b_{3}}{b_{2}}\frac{\left(1+\lambda \right)}{\left(1+\lambda_{2}s\right)}=\frac{\partial^{2}\bar{u}}{\partial y^{2}}-\frac{b_{1}}{b_{2}}s\bar{u}\left(y,s\right)\frac{\left(1+\lambda \right)}{\left(1+\lambda_{2}s\right)}$$

Substituting Equation (23) in (28), we have the following result.

(29) $$\bar{u}\left(y,s\right)=\left[\mathrm{Gr}\frac{b_{3}}{b_{2}}\frac{1}{\left(1+\lambda_{2}s\right)}\frac{1}{z(s)}\frac{\left(1+\lambda \right)}{s}\right]\left(e^{-y\sqrt{\frac{b_{1}\left(1+\lambda \right)}{b_{2}\left(1+\lambda_{2}s\right)}}}-e^{-y\sqrt{\frac{\mathrm{Pr}sb_{0}}{s^{\beta }{\left(1-as^{-\alpha }\right)}^{\gamma }}}}\right).$$

where

(30) $$z\left(s\right)=\frac{\mathrm{Pr}sb_{0}}{s^{\beta }{\left(1-as^{-\alpha }\right)}^{\gamma }}-\frac{b_{1}s\left(1+\lambda \right)}{b_{2}\left(1+\lambda_{2}s\right)}.$$

The inverse Laplace of Equation (29) can be obtained numerically by using Tzou’s and Stehfest’s algorithms [44,45].

## 4. Solution of Temperature and Velocity with Hybrid Nanoparticles

By following the same procedure of Section 3 solution of temperature with hybrid nanoparticles is given as

(31) $$\bar{\theta }\left(y,s\right)=s^{-1}e^{-y\sqrt{\frac{\mathrm{Pr}sc_{0}}{s^{\beta }{\left(1-as^{-\alpha }\right)}^{\gamma }}}}.$$

The inverse Laplace of Equation (31) is

$$\theta \left(y,t\right)=1+\sum_{m=1}^{\infty }\sum_{n=0}^{\infty }\frac{{\left(-y\right)}^{m}{\left(\mathrm{Pr}c_{0}\right)}^{\frac{m}{2}}}{m!}\frac{t^{\frac{\beta m-m}{2}+\alpha a}}{\Gamma (\frac{\beta m-m}{2}+\alpha a+1)}\frac{{\left(-a\right)}^{n}}{n!}\frac{\Gamma (\frac{-\gamma m}{2}+1)}{\Gamma (\frac{-\gamma m}{2}+1-n)}.$$

Solution of velocity with hybrid nanoparticles is given as

(32) $$\bar{u}\left(y,s\right)=\left[\mathrm{Gr}\frac{c_{3}}{c_{2}}\frac{\left(1+\lambda \right)}{\left(1+\lambda_{2}s\right)}\frac{1}{v(s)}\frac{1}{s}\right]\left(e^{-y\sqrt{\frac{c_{1}\left(1+\lambda \right)}{c_{2}\left(1+\lambda_{2}s\right)}}}-e^{-y\sqrt{\frac{\mathrm{Pr}sc_{0}}{s^{\beta }{\left(1-as^{-\alpha }\right)}^{\gamma }}}}\right).$$

where v, $c_{0}$, $c_{1}$, $c_{2}$ and $c_{3}$ are defined as $v\left(s\right)=\frac{\mathrm{Pr}sc_{0}}{s^{\beta }{\left(1-as^{-\alpha }\right)}^{\gamma }}-\frac{c_{1}s\left(1+\lambda \right)}{c_{2}\left(1+\lambda_{2}s\right)}$, $c_{0}=\left[\left(1-\left(\phi_{1}+\phi_{2}\right)\right)+\frac{\phi_{1}{\left(\rho C_{p}\right)}_{s_{1}}+\phi_{2}{\left(\rho C_{p}\right)}_{s_{2}}}{{\left(\rho C_{p}\right)}_{f}}\right]$, $c_{1}=\left[\left(1-\left(\phi_{1}+\phi_{2}\right)\right)+\frac{\phi_{1}\rho_{s_{1}}+\phi_{2}\rho_{s_{2}}}{\rho_{f}}\right]$, $c_{2}=\frac{1}{{\left(1-\left(\phi_{1}+\phi_{2}\right)\right)}^{2.5}}$, $c_{3}=\left[\left(1-\left(\phi_{1}+\phi_{2}\right)\right)+\frac{\phi_{1}{\left(\rho \beta_{T}\right)}_{s_{1}}+\phi_{2}{\left(\rho \beta_{T}\right)}_{s_{2}}}{\left(\rho \beta_{T}\right)}\right].$

Inverse Laplace of (32) can be obtained numerically by using Tzou’s and Stehfest’s algorithms [44,45].

## 5. Solution of Temperature and Velocity with Mono Nanoparticles

By following same procedure of Section 3 and Section 4 the temperature equation for mono nanoparticles is as follows:

(33) $$\bar{\theta }\left(y,s\right)=s^{-1}e^{-y\sqrt{\frac{\mathrm{Pr}sd_{0}}{s^{\beta }{\left(1-as^{-\alpha }\right)}^{\gamma }}}}.$$

In order to get the inverse Laplace transform of Equation (33), it can be written in the following form by using formula ${\left(1-x\right)}^{-p}=\sum_{q=0}^{\infty }x^{q}\left(\frac{\Gamma (p+q)}{q!}\left(\Gamma p\right)\right)$ [2].

The inverse Laplace of Equation (33) is

$$\theta \left(y,t\right)=1+\sum_{m=1}^{\infty }\sum_{n=0}^{\infty }\frac{{\left(-y\right)}^{m}{\left(\mathrm{Pr}d_{0}\right)}^{\frac{m}{2}}}{m!}\frac{t^{\frac{\beta m-m}{2}+\alpha a}}{\Gamma (\frac{\beta m-m}{2}+\alpha a+1)}\frac{{\left(-a\right)}^{n}}{n!}\frac{\Gamma (\frac{-\gamma m}{2}+1)}{\Gamma (\frac{-\gamma m}{2}+1-n)}.$$

Velocity field for mono nanoparticles is as follows:

(34) $$\bar{u}\left(y,s\right)=\left[\mathrm{Gr}\frac{d_{3}}{d_{2}}\frac{1}{\left(1+\lambda_{2}s\right)}\frac{1}{w(s)}\frac{\left(1+\lambda \right)}{s}\right]\left(e^{-y\sqrt{\frac{d_{1}\left(1+\lambda \right)}{d_{2}\left(1+\lambda_{2}s\right)}}}-e^{-y\sqrt{\frac{\mathrm{Pr}sd_{0}}{s^{\beta }{\left(1-as^{-\alpha }\right)}^{\gamma }}}}\right).$$

where w, $d_{0}$, $d_{1}$, $d_{2}$ and $d_{3}$ are defined as $w\left(s\right)=\frac{\mathrm{Pr}sd_{0}}{s^{\beta }{\left(1-as^{-\alpha }\right)}^{\gamma }}-\frac{d_{1}s\left(1+\lambda \right)}{d_{2}\left(1+\lambda_{2}s\right)}$, $d_{0}=\left[\left(1-\left(\phi_{1}\right)\right)+\frac{\phi_{1}{\left(\rho C_{p}\right)}_{s_{1}}}{{\left(\rho c_{p}\right)}_{f}}\right]$, $d_{1}=\left[\left(1-\left(\phi_{1}\right)\right)+\frac{\phi_{1}\rho_{s_{1}}}{\rho_{f}}\right]$, $d_{2}=\frac{1}{{\left(1-\left(\phi_{1}\right)\right)}^{2.5}}$, $d_{3}=\left[\left(1-\left(\phi_{1}\right)\right)+\frac{\phi_{1}{\left(\rho \beta_{T}\right)}_{s_{1}}}{{\left(\rho \beta_{T}\right)}_{f}}\right]$.

The inverse Laplace of Equation (34) can be obtained analytically by using Tzou’s and Stehfest’s algorithms [44,45].

## 6. Nusselt Numbers

### 6.1. Nusselt Number for Ternary Nanoparticles

The heat transfer rate in terms of the Nusselt number for ternary nanoparticles is given by the following relation (Table 3).

(35) $$Nu=-e_{1}k_{f}\frac{\partial \theta (0,t)}{\partial y}$$

where $e_{1}=\frac{\phi_{1}k_{1}+\phi_{2}k_{2}+\phi_{3}k_{3}+2\left(\phi_{1}+\phi_{2}+\phi_{3}\right)k_{f}+2\left(\phi_{1}+\phi_{2}+\phi_{3}\right)\left(\phi_{1}k_{1}+\phi_{2}k_{2}+\phi_{3}k_{3}\right)-2{\left(\phi_{1}+\phi_{2}+\phi_{3}\right)}^{2}k_{f}}{\phi_{1}k_{1}+\phi_{2}k_{2}+\phi_{3}k_{3}+2\left(\phi_{1}+\phi_{2}+\phi_{3}\right)k_{f}-\left(\phi_{1}+\phi_{2}+\phi_{3}\right)\left(\phi_{1}k_{1}+\phi_{2}k_{2}+\phi_{3}k_{3}\right)+{\left(\phi_{1}+\phi_{2}+\phi_{3}\right)}^{2}k_{f}}.$

### 6.2. Nusselt Number for Hybrid Nanoparticles

The heat transfer rate in terms of Nusselt number for hybrid nanoparticles is given by the following relation (Table 3).

(36) $$Nu=-e_{2}k_{f}\frac{\partial \theta (0,t)}{\partial y}$$

where $e_{2}=\frac{\phi_{1}k_{1}+\phi_{2}k_{2}+2\left(\phi_{1}+\phi_{2}\right)k_{f}+2\left(\phi_{1}+\phi_{2}\right)\left(\phi_{1}k_{1}+\phi_{2}k_{2}\right)-2{\left(\phi_{1}+\phi_{2}\right)}^{2}k_{f}}{\phi_{1}k_{1}+\phi_{2}k_{2}+2\left(\phi_{1}+\phi_{2}\right)k_{f}-\left(\phi_{1}+\phi_{2}\right)\left(\phi_{1}k_{1}+\phi_{2}k_{2}\right)+{\left(\phi_{1}+\phi_{2}\right)}^{2}k_{f}}.$

### 6.3. Nusselt Number for Mono Nanoparticles

The heat transfer rate in terms of the Nusselt number for mono nanoparticles is given in the following relation (Table 3).

(37) $$Nu=-e_{3}k_{f}\frac{\partial \theta (0,t)}{\partial y}$$

where $e_{3}=\frac{\phi_{1}k_{1}+2\left(\phi_{1}\right)k_{f}+2\left(\phi_{1}\right)\left(\phi_{1}k_{1}\right)-2{\left(\phi_{1}\right)}^{2}k_{f}}{\phi_{1}k_{1}+2\left(\phi_{1}\right)k_{f}-\left(\phi_{1}\right)\left(\phi_{1}k_{1}\right)+{\left(\phi_{1}\right)}^{2}k_{f}}.$

## 7. Skin Friction

### 7.1. Skin Friction for Ternary Nanoparticles

The skin friction for ternary nanaoparticles is given in the following relation (Table 4).

(38) $$C_{f}=f_{1}\mu_{f}\frac{\partial u(0,t)}{\partial y}$$

where $f_{1}=\frac{1}{{\left(1-\left(\phi_{1}+\phi_{2}+\phi_{3}\right)\right)}^{2.5}}.$

### 7.2. Skin Friction for Hybrid Nanoparticles

The skin friction relation for hybrid nanaoparticles is given by (Table 4).

(39) $$C_{f}=f_{2}\mu_{f}\frac{\partial u(0,t)}{\partial y}$$

where $f_{2}=\frac{1}{{\left(1-\left(\phi_{1}+\phi_{2}\right)\right)}^{2.5}}.$

### 7.3. Skin Friction for Mono Nanoparticles

The skin friction relation for mono nanoparticels is given by (Table 4).

(40) $$C_{f}=f_{3}\mu_{f}\frac{\partial u(0,t)}{\partial y}$$

where $f_{3}=\frac{1}{{\left(1-\phi_{1}\right)}^{2.5}}.$

## 8. Graphical Results and Discussion

The prevailing work deals with the Jeffrey fluid moving along a vertical plate. Laplace transform is applied to obtain an analytical solution. The graphical investigation is made by means of Mathcad software.

**Figure 2:**
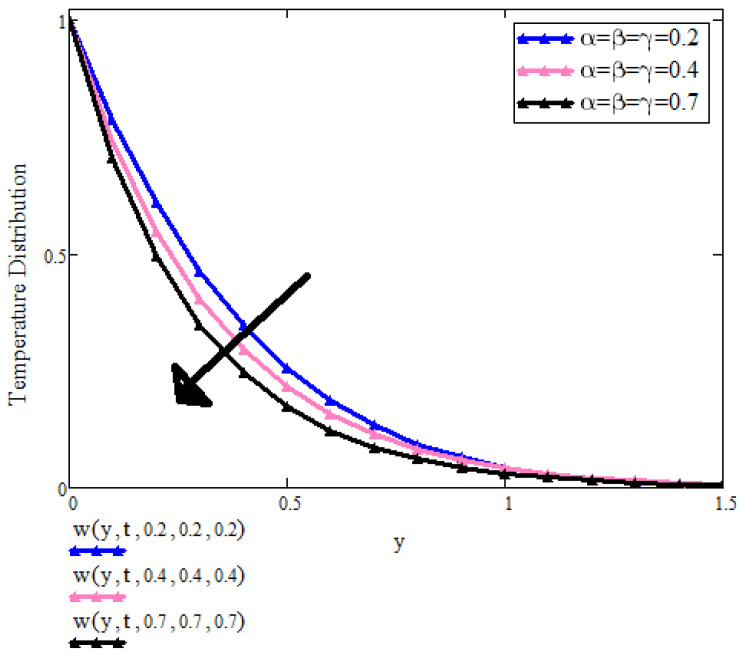
T(y,t) with α,β,γ when t=0.8, a=0.9, λ=0.5, Pr=6.2, $\beta_{1}=0.9$, $\phi_{1}=\phi_{2}=\phi_{3}=0.03$.

Figure 2 shows the effect of fractional parameters on the temperature profile. It can be seen that by increasing the values of fractional parameters α, β, γ, the temperature is decreasing. Physically, the fractional solution behaves as dual for small and large times for different values of fractional parameters. It is evident that for large values of time, the fluid temperature decreases. Therefore, we have a choice to achieve the desired results of the fractional model.

**Figure 3:**
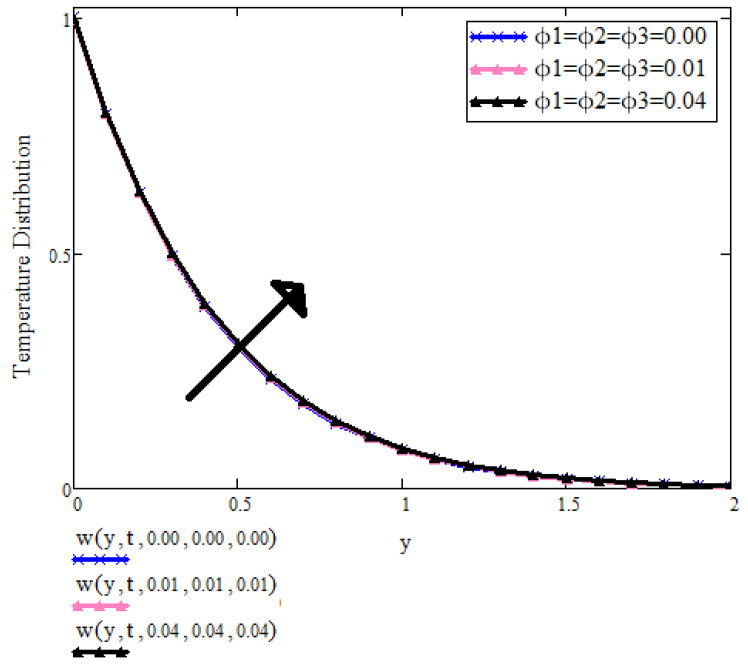
T(y,t) with $\phi_{1},\phi_{2},\phi_{3}$ when t=1, a=0.3, λ=0.7, Pr=6.2, $\beta_{1}=0.5$.

Figure 3 is showing the influence of the concentration of nanoparticles $\phi_{1},\phi_{2},\phi_{3}$ on the temperature field. It is observed that with increasing the concentration of nanoparticles that the temperature is also increasing. Physically, increasing the concentration of nanoparticles leads to increase thermal conductivity and ultimately the thickness of thermal conductivity and hence temperature increases.

**Figure 4:**
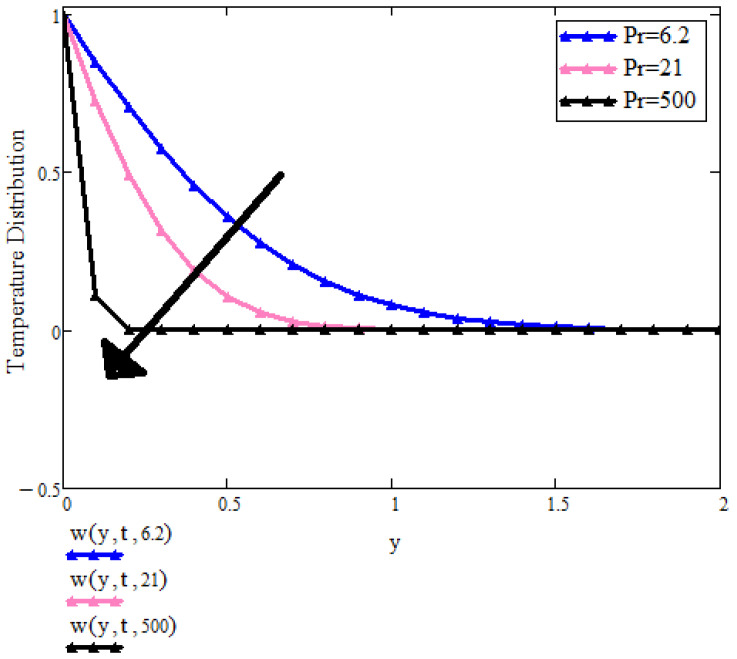
T(y,t) with Pr when t=1, a=0.5, λ=0.1, Pr=6.2, $\beta_{1}=0.1$a, $\phi_{1}=\phi_{2}=\phi_{3}=0.03$.

Figure 4 is showing the behavior of Pr with respect to the temperature field. When Pr values increase, temperature distribution declines. Pr number is a ratio between momentum diffusivity to heat diffusivity. Fluids with inferior Pr number values have greater thermal conductivity, which causes heat diffuse far from the heated surface more swiftly and quicker in comparison to increased values of Pr. Consequently, Figure 4 represents the ternary nanoparticles by choosing different base fluids, for example water, kerosene, and engine oil, respectively. It is very clear that by taking engine oil as the base fluid, the temperature decreases due to high values of Prandtl number.

**Figure 5:**
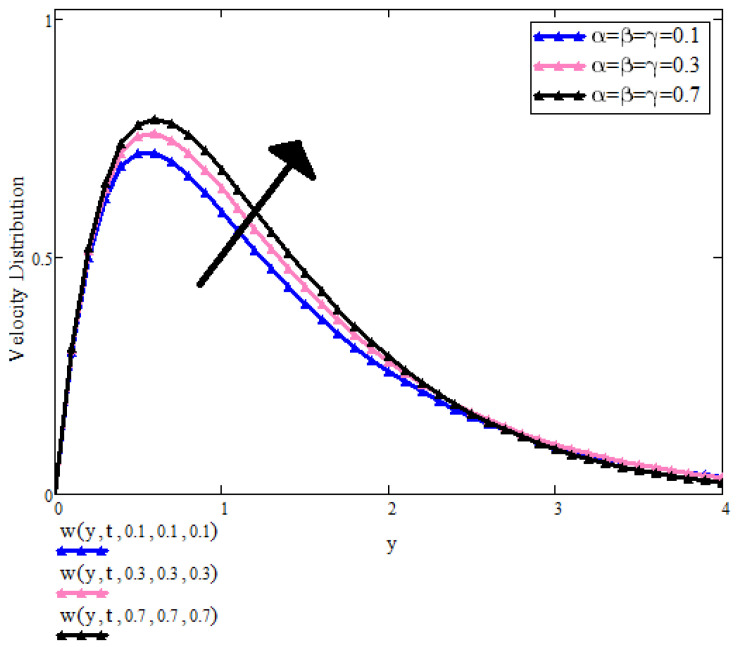
u(y,t) with α,β,γ when t=1, a=0.1, λ=0.5, $\lambda_{2}=0.9$, Pr=6.2, $\beta_{1}=0.9$, $\phi_{1}=\phi_{2}=\phi_{3}=0.05$.

Figure 5 shows the impact of fractional parameters on the velocity profile. It can be seen that by increasing the values of fractional parameters, the velocity profile increases. With time, the boundary layer thickness increases and the velocity is extreme in that area of the plate. Physically, the fractional solution behaves dual for small and large times for different values of fractional parameters. It is evident that for large values of time, the fluid velocity increases. Therefore, we have a choice to achieve the desired results of the fractional model.

**Figure 6:**
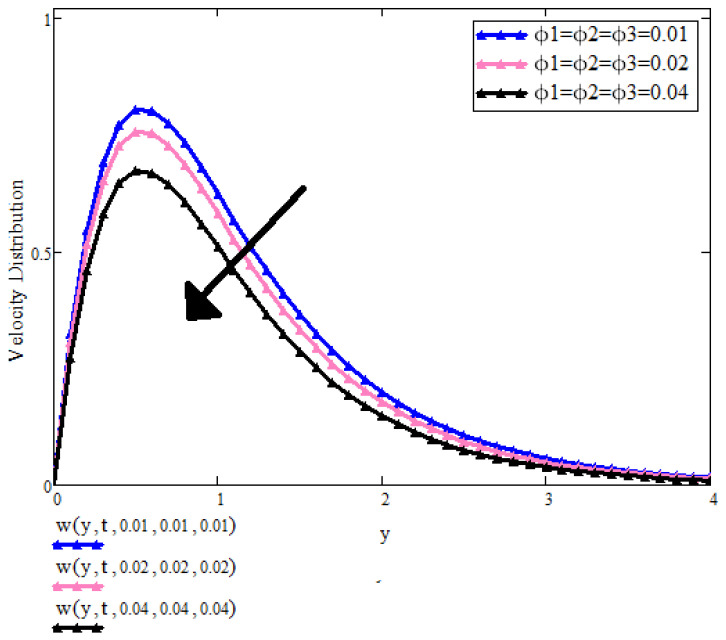
u(y,t) with $\phi_{1},\phi_{2},\phi_{3}$ when t=1, a=0.01, λ=0.5, $\lambda_{2}=0.9$, Pr=6.2, $\beta_{1}=0.9$, $\phi_{1}=\phi_{2}=\phi_{3}=0.02$.

Figure 6 is intended to show the effect of the concentration of nanoparticles on the velocity field. It can be seen that by increasing the concentration of nanoparticles velocity profile is decreasing. Figure 6 depicted the impact of the concentration of ternary nanoparticles on velocity. It is found that larger the values of $\phi_{1},\phi_{2}$ and $\phi_{3}$ stronger the viscous force makes the boundary layer denser and hence decreases.

**Figure 7:**
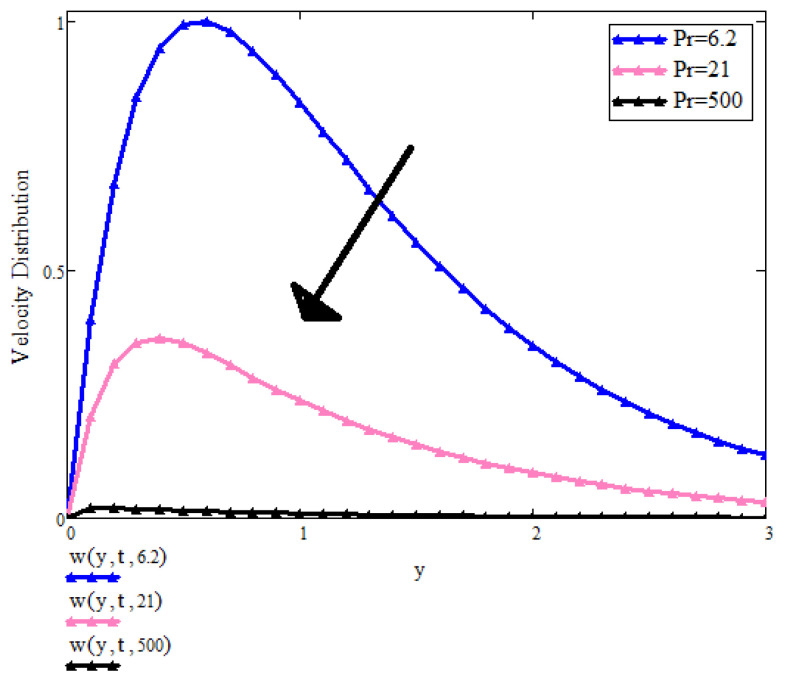
u(y,t) with Pr when t=1.2, a=0.05, λ=0.5, $\lambda_{2}=0.9$, Pr=6.2, $\beta_{1}=0.9$, $\phi_{1}=\phi_{2}=\phi_{3}=0.05$.

Figure 7 shows the effect of Pr on the velocity field. By raising Pr values, the velocity distribution is decreasing. Due to the difference between the thermal conductivities of different base fluids, the thermal conductivity of water is comparatively higher than kerosene and engine oil. Velocity became higher, and a more distinctive peak is observed near the plate. Therefore, an upsurge in the thickness of the boundary layer condenses velocity distribution.

**Figure 8:**
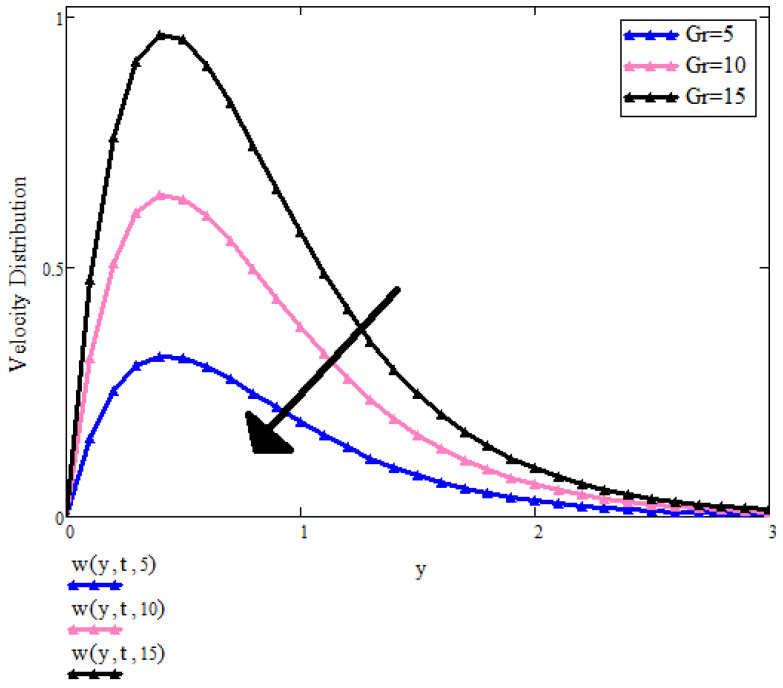
u(y,t) with Gr when t=1, a=0.9, λ=0.5, $\lambda_{2}=0.9$, Pr=6.2, $\beta_{1}=0.9$, $\phi_{1}=\phi_{2}=\phi_{3}=0.05$.

Figure 8 is indicating the effect of Grashof number Gr on the velocity profile. The velocity of the fluid is increasing by increasing Gr values. Gr number is a ratio between buoyancy forces and viscous forces. For increased Gr values, buoyancy forces increase, which upsurges the induced flow as an outcome, and the velocity of the fluid can be enhanced.

**Figure 9:**
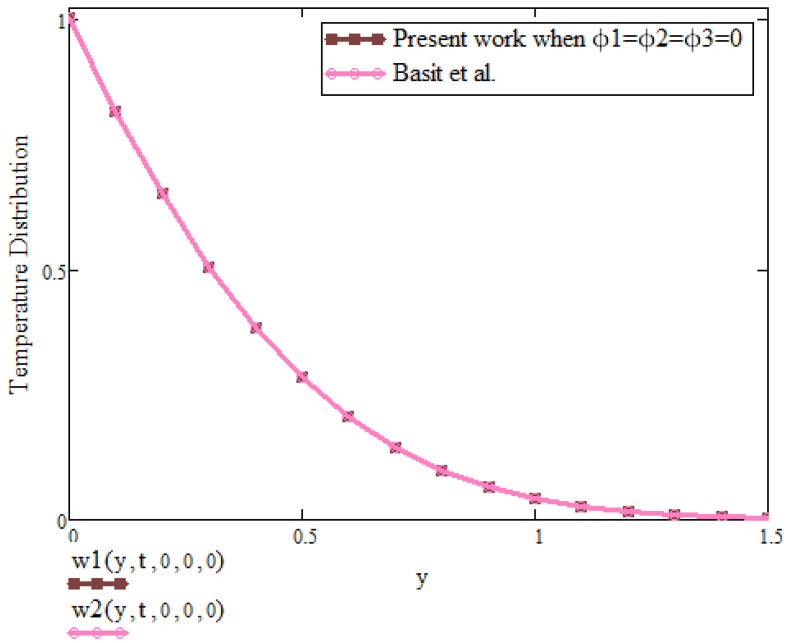
Comparison graph of temperature between present work and Basit et al. [2] when t=0.8, a=0.9, λ=0.5, Pr=6.2, $\beta_{1}=0.9$.

Figure 9 is about the comparison of temperature between present result and already published work by Basit et al. [2]. From the figure, it is clear that in the absence of concentration of nanoparticles $\phi_{1},\phi_{2},\phi_{3}$ = 0, the result from recent literature are retrieved. This fact provides the validation of the present work.

**Figure 10:**
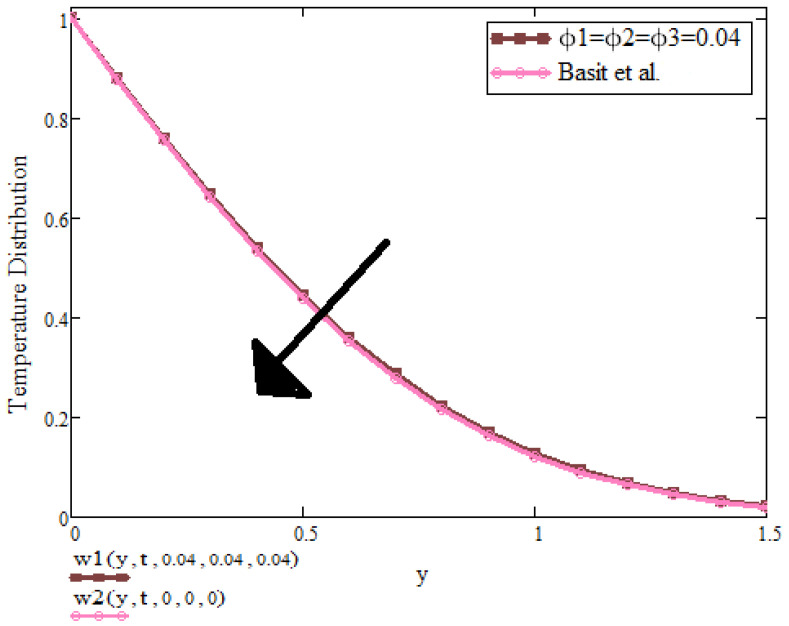
T(y,t) with $\phi_{1},\phi_{2},\phi_{3}$ when t=1.3, a=0.4, λ=1, Pr=6.2, $\beta_{1}=0.9$ [2].

Figure 10 shows that temperature distribution declines in the presence of nanoparticles. It is found that the presence of ternary nanoparticles can be used to enhance the temperature.

**Figure 11:**
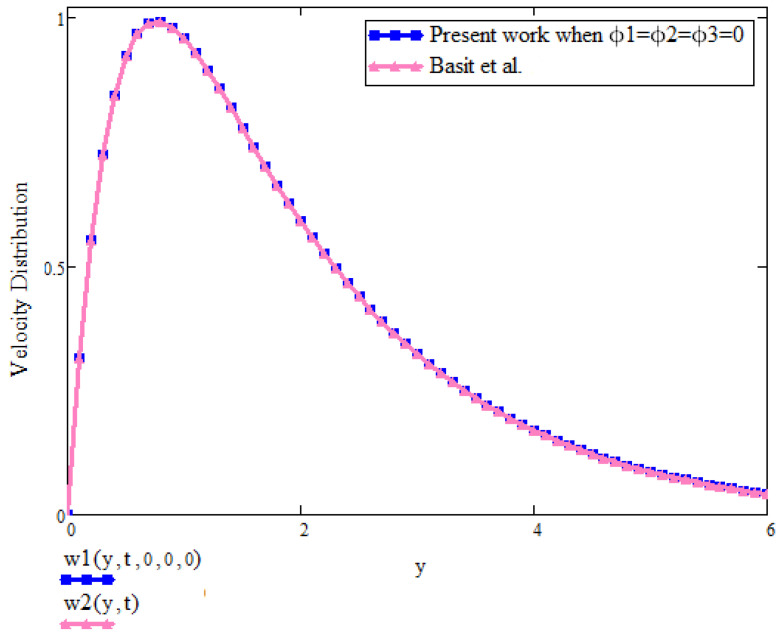
Comparison graph of velocity between present work and Basit et al. [2] when t=2, a=0.1, λ=0, Gr=15, $\beta_{1}=0.9$.

Figure 11 is about the comparison of velocity between the present result and already published work by Basit et al. [2]. From the figure, it is clear that in the absence of concentration of nanoparticles $\phi_{1},\phi_{2},\phi_{3}$ = 0, the results from recent literature are retrieved. This fact provides the validation of the present work.

**Figure 12:**
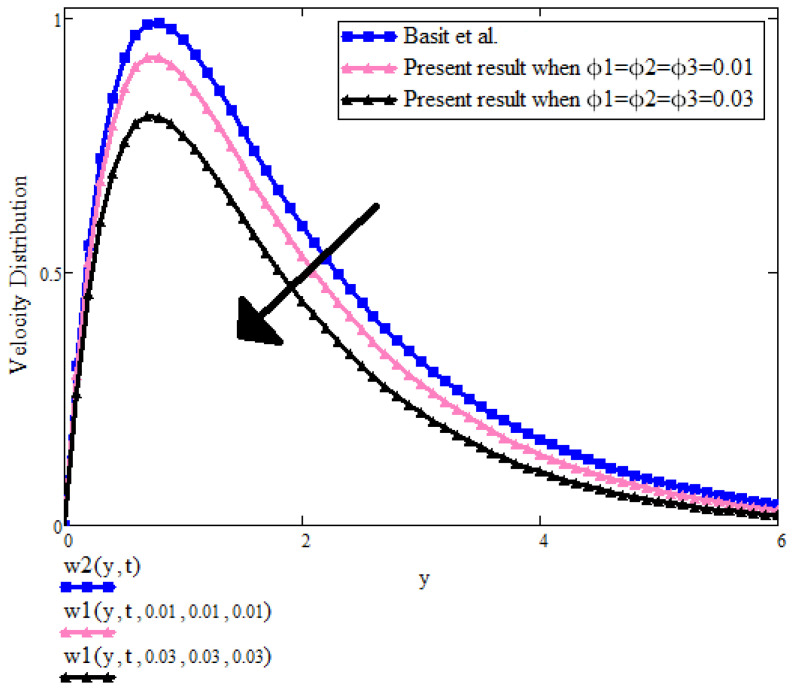
u(y,t) with $\phi_{1},\phi_{2},\phi_{3}$ when t=2, a=0.1, λ=0, Gr=15, $\beta_{1}=0.9$ [2].

Figure 12 is also the comparison of velocity with and without the presence of nanoparticles. It is found that velocity in the absence of ternary nanoparticles velocity is higher. This is due to the fact that fluid becomes less thick and the distance between boundary layers increases and boosts up the flow.

To see the influence of fractional parameters and time on the rate of heat transfer and skin friction, we have constructed Table 3 and Table 4. From Table 3, it can be seen that the heat transfer rate in terms of the Nusselt number is decreasing by enhancing fractional parameter values and increasing with respect to time. On further notice, it is observed that heat transfer for ternary nanoparticles is greater as compared to hybrid and mono nanoparticles. Therefore, this approach can be used in practical situations, especially in heat management systems and electronic appliances. Similarly, from Table 4, we can see that the effect of fractional parameter α on the skin friction is estimated numerically and indicates that the skin friction is an increasing function when values of α are increased. Additionally, it is observed that skin friction for ternary nanoparticles is greater in comparison to hybrid and mono nanoparticles, respectively.

## 9. Conclusions

In this paper, the unsteady flow of a Jeffrey fluid moving along a vertically placed plate is resolved analytically with the help of the Laplace transform. Effects of fractional parameters, the concentration of nanoparticles, Prandtl number Pr and thermal Grashof number Gr on temperature as well as velocity distribution are obtained graphically. The main results of this paper are as follows:

1. The fractional and ternary nanoparticles approach is helpful in improving the exiting results.
2. Choosing a specific value of fractional parameters, thermal and momentum boundary layers can be controlled for different time. Such a fractional approach is very helpful in handling the experimental data by using theoretical information.
3. The rate of heat transfer for ternary nanoparticles is greater in comparison of hybrid and mono nanoparticles, respectively.
4. The obtained results are also useful in the sense of choosing base fluids (water, kerosene and Engin oil) for three types of naoparticles to achieved the desired targets in any transport problem.
5. The new approach with ternary nanoparticles is more reliable and effective, and was validated with the existing literature.

## Figures and Tables

**Table 1:** Thermodynamics properties of nanomaterials.

Thermodynamics Properties	ρ (kgm${}^{3}$)	$C_{p}$ (Jkg${}^{-1}$K${}^{-1}$)	*K* (Wm${}^{-1}$K${}^{-1}$)	σ (s/m)
Titanium oxide ($TiO_{2}$)	4250	686.2	8.9538	$10^{-9}-10^{11}$
Sliver (Ag)	10,500	235	418	6.3 $\times \;10^{7}$
Base fluid	997.1	4179	0.163	0.05
Aluminum oxide $\left(Al_{2}0_{3}\right)$	3970	765	40	35 $\times \;10^{6}$

**Table 2:** Properties of mono, hybrid, and ternary nanoparticles [12].

**Ternary nanofluid**
$u_{mnf}=u_{f}{\left(1-\left(\phi_{1}+\phi_{2}+\phi_{3}\right)\right)}^{-2.5}$
$\rho_{mnf}=p_{f}\left(1-\left(\phi_{3}+\phi_{1}+\phi_{2}\right)\right)+\phi_{3}\rho_{s_{3}}+\phi_{1}\rho_{s_{1}}+\phi_{2}\rho_{s_{2}}$
${\left(\rho C_{p}\right)}_{mnf}={\left(\rho C_{p}\right)}_{f}\left(1-\left(\phi_{3}+\phi_{1}+\phi_{2}\right)\right)+\phi_{2}{\left(\rho C_{p}\right)}_{s_{2}}+\phi_{1}{\left(\rho C_{p}\right)}_{s_{1}}+\phi_{3}{\left(\rho C_{p}\right)}_{s_{3}}$
${\left(\rho \beta_{T}\right)}_{mnf}={\left(\rho \beta_{T}\right)}_{f}\left(1-\left(\phi_{3}+\phi_{1}+\phi_{2}\right)\right)+\phi_{2}{\left(\rho \beta_{T}\right)}_{s_{2}}+\phi_{1}{\left(\rho \beta_{T}\right)}_{s_{1}}+\phi_{3}{\left(\rho \beta_{T}\right)}_{s_{3}}$
**Hybrid nanoparticles**
$u_{hnf}=u_{f}{\left(1-\left(\phi_{2}+\phi_{1}\right)\right)}^{-2.5}$
$\rho_{hnf}=p_{f}\left(1-\left(\phi_{2}+\phi_{1}\right)\right)+\phi_{2}\rho_{s_{2}}+\phi_{1}\rho_{s_{1}}$
${\left(\rho C_{p}\right)}_{hnf}={\left(\rho C_{p}\right)}_{f}\left(1-\left(\phi_{2}+\phi_{1}\right)\right)+\phi_{2}{\left(\rho C_{p}\right)}_{s_{2}}+\phi_{1}{\left(\rho C_{p}\right)}_{s_{1}}$
${\left(\rho \beta_{T}\right)}_{hnf}={\left(\rho \beta_{T}\right)}_{f}\left(1-\left(\phi_{2}+\phi_{1}\right)\right)+\phi_{2}{\left(\rho \beta_{T}\right)}_{s_{2}}+\phi_{1}{\left(\rho \beta_{T}\right)}_{s_{1}}$
**Mono nanoparticles**
$u_{nf}=u_{f}{\left(1-\left(\phi_{1}\right)\right)}^{-2.5}$
$\rho_{nf}=p_{f}\left(1-\left(\phi_{1}\right)\right)+\phi_{2}\rho_{s_{2}}+\phi_{1}\rho_{s_{1}}$
${\left(\rho C_{p}\right)}_{nf}={\left(\rho C_{p}\right)}_{f}\left(1-\left(\phi_{1}\right)\right)+\phi_{1}{\left(\rho C_{p}\right)}_{s_{1}}$
${\left(\rho \beta_{T}\right)}_{nf}={\left(\rho \beta_{T}\right)}_{f}\left(1-\left(\phi_{1}\right)\right)+\phi_{1}{\left(\rho \beta_{T}\right)}_{s_{1}}$

**Table 3:** Effect of time and fractional parameter on Nusselt number (ternary, hybrid and mono nanoparticles).

α,β,γ	Nu for Ternary		Nu for Hybrid		Nu for Mono	
	t=0.1	t=0.3	t=0.1	t=0.3	t=0.1	t=0.3
0.4	3.123	2.466	2.958	2.356	2.802	2.213
0.5	2.92	2.46	2.765	2.33	2.62	2.207
0.6	2.704	2.418	2.561	2.391	2.426	2.17
0.7	2.486	2.352	2.355	2.227	2.231	2.11
0.8	2.275	2.267	2.155	2.148	2.042	2.035
0.9	2.075	2.173	1.963	2.058	1.861	1.949
1	1.886	2.072	1.768	1.962	1.692	1.859

**Table 4:** Effect of time and fractional parameter on skin friction (Ternary, Hybrid and Mono nanoparticles).

α,β,γ	$C_{f}$ for Ternary		$C_{f}$ for Hybrid		$C_{f}$ for Mono	
	t=0.1	t=0.3	t=0.1	t=0.3	t=0.1	t=0.3
0.4	0.325	1.249	0.312	1.247	0.299	1.183
0.5	0.379	1.45	0.377	1.447	0.359	1.363
0.6	0.469	1.727	0.467	1.721	0.441	1.602
0.7	0.698	2.327	0.6	2.127	0.559	1.943
0.8	0.845	2.822	0.825	2.812	0.751	2.481
0.9	1.299	4.33	1.293	4.274	1.123	3.492
1	2.375	12.934	2.34	12.227	1.975	6.693

## Data Availability

All the data are given within the article.

## Nomenclature

Symbol	Names
ρ	Density of fluid
$C_{p}$	Heat capacity of fluid
μ	Viscosity of fluid
*G*	Gravitational acceleration
Pr	Prandtl number
ν	Kinematic viscosity
*t*	Time
$T_{w}$	Temperature at plate
$T_{\infty }$	Temperature at infinity
*k*	Thermal conductivity
$\beta_{T}$	Thermal expansion
α,β,γ	Fractional parameters
${\left(\rho C_{p}\right)}_{s_{1}},{\left(\rho C_{p}\right)}_{s_{2}},{\left(\rho C_{p}\right)}_{s_{3}}$	Specific heat for solid nanoparticles of type one, two, three
${\left(\rho \beta_{T}\right)}_{s_{1}},{\left(\rho \beta_{T}\right)}_{s_{2}},{\left(\rho \beta_{T}\right)}_{s_{3}}$	Thermal heat expansion for temperature one, two, three
$\phi_{1},\phi_{2},\phi_{3}$	Solid volume fraction of type one, two, three
a	Constant between zero and one
